# Supplementary figures and images for: Autopsy Study Defines Composition and Dynamics of the HIV-1 Reservoir after Allogeneic Hematopoietic Stem Cell Transplantation with CCR5Δ32/Δ32 Donor Cells
Source: Viruses. 2022 Sep 17;14(9):2069. doi: 10.3390/v14092069 (PMC9503691; doi:10.3390/v14092069)

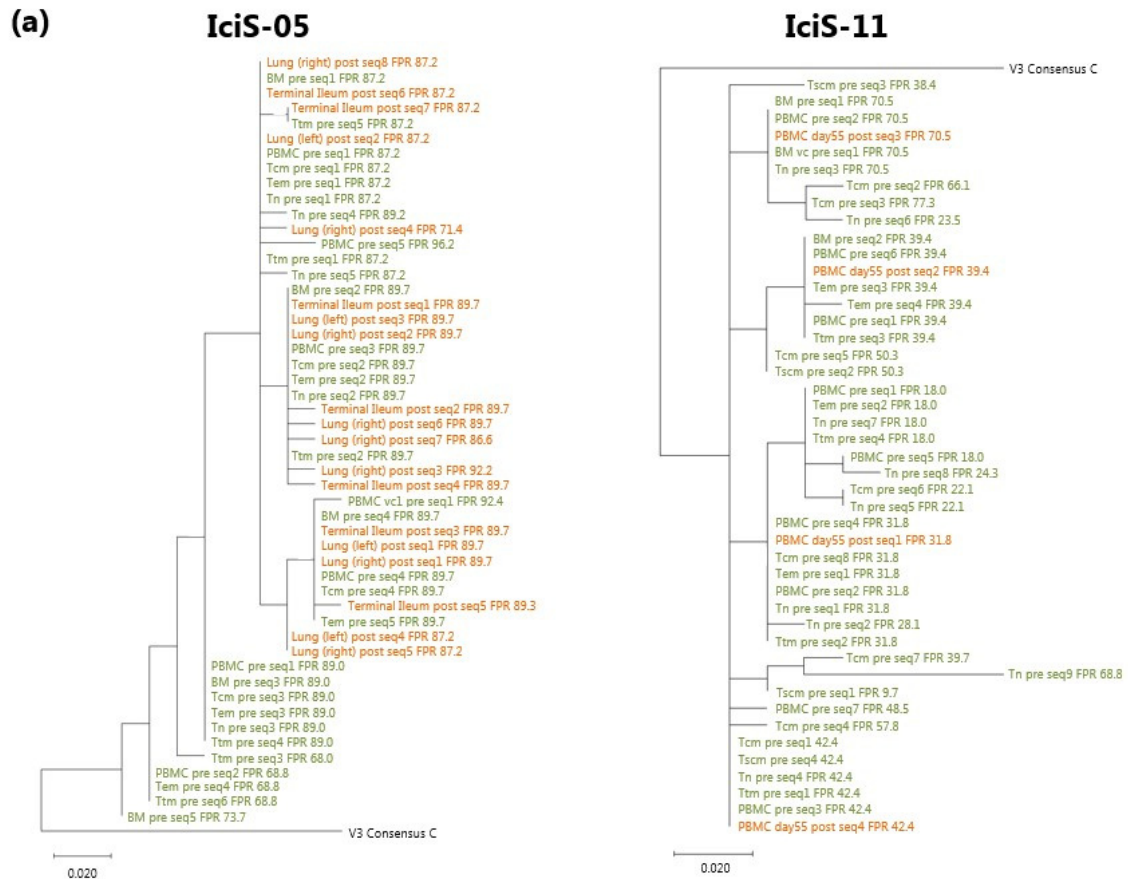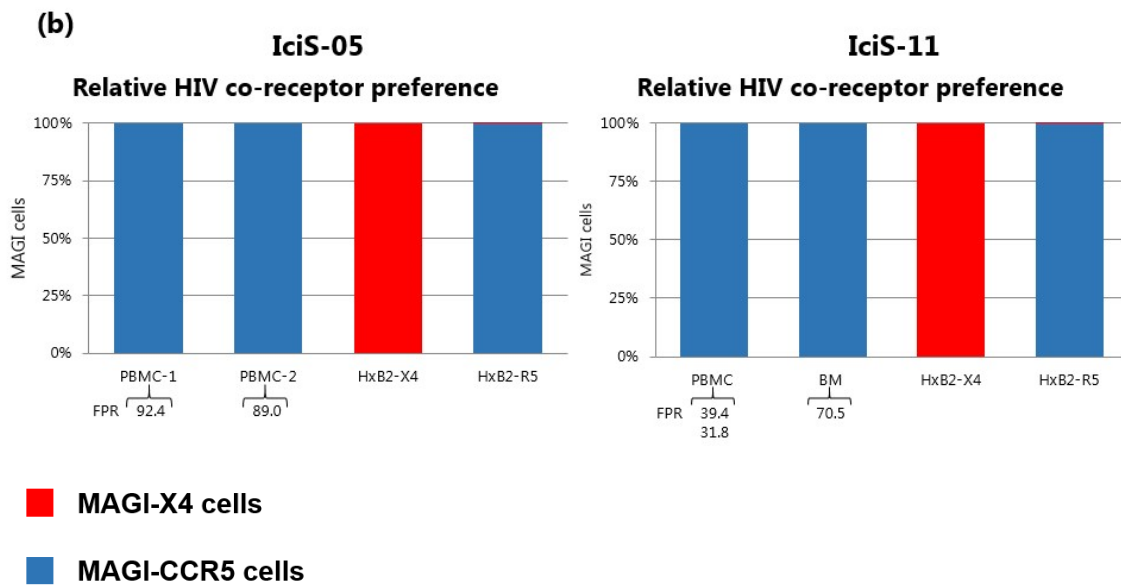

Figure S1: Phylogenetic analysis and relative coreceptor preference.

Supplement: Supplementary file 1 [file viruses-14-02069-s001.zip › viruses-1917284-supplementary.pdf]
